# Tomato-Poisoning

**Published:** 1890-04

**Authors:** William A. Mills

**Affiliations:** Baltimore, Md.


### TOMATO-POISONING.[1]

BY WILLIAM A. MILLS, D.D.S., BALTIMORE, MD.

Gentlemen,—I desire in this paper to call the attention of the profession to a new disease, which, after many years of experience, I have discovered to be and have named tomato-poisoning.

Of necessity I shall be brief in details, leaving the history of its discovery for some future paper; nor will I attempt any explanation of its pathology, but will leave it to others to philosophize upon its phenomena.

*Symptoms.*—At first the pain is rather indistinct, only manifesting itself in an uneasy discomfortable feeling, being more pronounced as evening approaches, and most acute at night. These symptoms may continue for days or weeks before the patient seeks relief from the agonizing pain which follows. Pain is rarely ever localized, but is made manifest by all kinds of reflex conditions. Patient cannot take anything sweet, even moderately warm or cold, into the mouth without causing the most intense agony. Going from the house into the open air, or from one room to another, where there is but a slight difference of temperature, produces the most acute paroxysm; sometimes seeming to proceed from an exposed or inflamed nerve, centring in one or more teeth in the same maxilla, or both; then apparently jumping from tooth to tooth, and then, by reflex action, causing neuralgia of the neck, head, ear, or angles of the jaws, alternating from point to point with the swiftness of the electric current. The duration and intensity are governed by age and temperament of the patient.

*Diagnosis.*—Upon the most careful examination of the oral cavity we fail to find any decay in the tooth the patient complains of, nor in any others in which reflex action could be supposed to play any part in the distressing symptoms. No response to concussion, no deposit of any kind, on any part of the teeth. All indications of pyorrhœa alveolaris absent, rarely any devitalized teeth; in general, the gums and associated parts apparently normal; mouth clean and properly attended.

---

[1] Read before the Maryland State Dental Association, December 5, 1889.

But, on very close examination, we discover around the necks of the superior molars (but rarely the inferior), particularly manifest on the palatine surfaces,—and even recession of the gums, about an eighth of an inch wide, as even and clean cut as though done with the most accurate mathematical precision,—the denuded surfaces are clean and white, in striking contrast with the surfaces above the line of original attachment of the gums.

If we place a steel instrument anywhere along the line of the recession, touch it with the finger-nail, or blow air upon it from the syringe, the patient exclaims in agony. So far all remedies have failed to effect a cure except the absolute prohibition of tomatoes as an article of food. All the foregoing pertains only to patients from eighteen to twenty-five years of age. With older patients the disease proves more serious. In its first stages all the symptoms of the younger are manifest, lasting about two or three weeks, when the tissue surrounding the tooth or teeth affected shrink again to their normal position, but do not become attached to the teeth as in the case of the younger. About the fourth week the gums surrounding the necks of the teeth show a slight thickening of tissue, the color of which resembles the line of lead-poisoning. The slightest pressure of the tongue causes great pain, apparently deep-seated in the maxilla; the sensation imparted to the tongue, when touched by it, is that of something foreign and glassy. About the fifth week the teeth begin to loosen, the alveolar wall is absorbed, and in some cases caries of the roots takes place. About the sixth week the tissues collapse, when we have a condition resembling that of cancrum oris or arsenious-acid poisoning.

At this point the disease has reached its climax. If the tooth does not fall out from lost attachment, it must be extracted to give relief, which will be but slight, as all pain does not cease for days afterwards. Prohibition of tomatoes gives no relief in these cases; all remedies have failed to give even temporary relief, nothing but extraction effecting a cure.

Patients rarely lose more than one tooth the same year. The disease is most prevalent during the wet seasons. This is no doubt due to the greater amount of acid contained in the tomatoes. The disease never attacks a devitalized tooth. One great peculiarity about the disease is that, during the whole process of absorption, there is almost a total absence of fœtor. In diagnosticating this disease do not confound its symptoms with that produced by grape and tomato sore mouth; always hunt for the recession of the gums on the palatine surfaces of the superior molars.

